# Type 1 diabetes: Developing the first risk-estimation model for predicting silent myocardial ischemia. The potential role of insulin resistance

**DOI:** 10.1371/journal.pone.0174640

**Published:** 2017-04-03

**Authors:** Gemma Llauradó, Albert Cano, Cristina Hernández, Montserrat González-Sastre, Ato-Antonio Rodríguez, Jordi Puntí, Eugenio Berlanga, Lara Albert, Rafael Simó, Joan Vendrell, José-Miguel González Clemente

**Affiliations:** 1 Department of Endocrinology and Nutrition, Hospital del Mar, Barcelona, Spain; 2 Centro de Investigación Biomédica en Red de Diabetes y Enfermedades Metabólicas Asociadas (CIBERDEM), Instituto de Salud Carlos III, Madrid, Spain; 3 Department of Endocrinology and Nutrition, Parc Taulí Hospital Universitari, Institut d’Investigació i Innovació Parc Taulí I3PT, Universitat Autònoma de Barcelona, Sabadell, Spain; 4 Diabetes and Metabolism Research Unit, Institut de Recerca Hospital Universitari Vall d‘Hebron, Universitat Autònoma de Barcelona, Barcelona, Spain; 5 Ophthalmology Department, Parc Taulí Hospital Universitari, Institut d’Investigació i Innovació Parc Taulí I3PT, Universitat Autònoma de Barcelona, Sabadell, Spain; 6 Nuclear Medicine Department, UDIAT Centre Diagnòstic, Institut d’Investigació i Innovació Parc Taulí I3PT, Universitat Autònoma de Barcelona, Sabadell, Spain; 7 Cardiology Department, Parc Taulí Hospital Universitari, Institut d’Investigació i Innovació Parc Taulí I3PT, Universitat Autònoma de Barcelona, Sabadell, Spain; 8 Biochemistry Department, UDIAT Centre Diagnòstic, Institut d’Investigació i Innovació Parc Taulí I3PT, Universitat Autònoma de Barcelona, Sabadell, Spain; 9 Hospital Universitari Joan XXIII de Tarragona, Institut d’Investigacions Sanitàries Pere Virgili (IISPV), Universitat Rovira i Virgili, Tarragona, Spain; University of Adelaide, AUSTRALIA

## Abstract

**Objectives:**

The aim of the study was to develop a novel risk estimation model for predicting silent myocardial ischemia (SMI) in patients with type 1 diabetes (T1DM) and no clinical cardiovascular disease, evaluating the potential role of insulin resistance in such a model. Additionally, the accuracy of this model was compared with currently available models for predicting clinical coronary artery disease (CAD) in general and diabetic populations.

**Research, design and methods:**

Patients with T1DM (35–65years, >10-year duration) and no clinical cardiovascular disease were consecutively evaluated for: 1) clinical and anthropometric data (including classical cardiovascular risk factors), 2) insulin sensitivity (estimate of glucose disposal rate (eGDR)), and 3) SMI diagnosed by stress myocardial perfusion gated SPECTs.

**Results:**

Eighty-four T1DM patients were evaluated [50.1±9.3 years, 50% men, 36.9% active smokers, T1DM duration: 19.0(15.9–27.5) years and eGDR 7.8(5.5–9.4)mg·kg^-1^·min^-1^]. Of these, ten were diagnosed with SMI (11.9%). Multivariate logistic regression models showed that only eGDR (OR = -0.593, p = 0.005) and active smoking (OR = 7.964, p = 0.018) were independently associated with SMI. The AUC of the ROC curve of this risk estimation model for predicting SMI was 0.833 (95%CI:0.692–0.974), higher than those obtained with the use of currently available models for predicting clinical CAD (Framingham Risk Equation: 0.833 vs. 0.688, p = 0.122; UKPDS Risk Engine (0.833 vs. 0.559; p = 0.001) and EDC equation: 0.833 vs. 0.558, p = 0.027).

**Conclusion:**

This study provides the first ever reported risk-estimation model for predicting SMI in T1DM. The model only includes insulin resistance and active smoking as main predictors of SMI.

## Introduction

Cardiovascular disease (CVD) is the main cause of death in patients with type 1 diabetes mellitus (T1DM)[1], representing around 40–47% of deaths in certain cohorts [2,3]. Coronary artery disease (CAD) is its principal clinical manifestation [4]. The relative risk of death by CAD in T1DM can be as much as ten times greater than in the non-diabetic population, especially in women, and it is even greater than the relative risk in type 2 diabetes (T2DM)[1,5]. It causes a life-expectancy loss of about four years, which represents one-third of these subjects’ total life-expectancy loss [6]. Additionally, CAD produces important disabilities (e.g., heart failure, angina), which cause quality of life to deteriorate and involve considerable economic costs.

In T1DM, CAD is usually diagnosed in advanced stages and is associated with a worse prognosis compared with the non-diabetic population [7]. This delayed diagnosis is partially explained by a higher prevalence of silent myocardial ischemia (SMI), being silent approximately half of all the myocardial infarctions [8].

SMI is defined as the presence of myocardial ischemia without symptoms and it is usually present long before the first CAD event occurs. The prevalence of SMI in subjects with T1DM is in the range 15–43% [9–11], while it affects 1–4% of non-diabetic subjects [12]. Its presence is associated with a worse prognosis and it predicts major cardiovascular events [13–15]. Therefore, early identification of SMI in subjects with T1DM is essential. However, performing a screening test for SMI in the whole T1DM population is unfeasible (both from a clinical and economic point of view), making such identification a real challenge.

The recent Scientific Statement from the American Heart Association (AHA) and the American Diabetes Association (ADA) on cardiovascular disease in T1DM discourages routine CAD screening beyond resting ECG [4]. The ADA/AHA guidelines recommend performing additional testing for CAD on any patient (including those with T1DM) who has symptoms, an abnormal resting ECG or a clustering of cardiovascular risks factors that yields an intermediate/high risk (based on general algorithms)[4]. However, it is also pointed out that neither general (Framingham) nor T2DM (UKPDS) risk algorithms are good enough for risk prediction in T1DM, suggesting the use of models specifically obtained from T1DM cohorts (such as the Pittsburgh Epidemiology of Diabetes Complications Study (EDC) cohort)[4]. Finally, there is an urgent need to find novel risk-estimation tools for better prediction of cardiovascular events in T1DM [4].

The aim of the current study was to develop a novel, specific SMI-risk estimation model to identify those patients with T1DM at highest risk of SMI as the initial step in improving prevention, treatment and prognosis of CAD events. To this end, we evaluated 84 patients with T1DM, aged 35–65 years old, with at least 10-year illness duration and no previous clinical cardiovascular disease and consecutively recruited at an outpatient clinic. As, in T1DM, insulin resistance has been associated with incident cardiovascular disease, as shown in the DCCT/EDIC, the Pittsburgh EDC and the FinnDiane studies [16–19], we additionally hypothesized that it might play a key role in identifying patients at highest risk of SMI. Finally, we examined the accuracy of current models for predicting clinical CAD (Framingham, UKPDS and EDC Study) in the prediction of SMI in our population.

## Materials and methods

### Study subjects

Eighty-four patients aged 35–65 years, with T1DM of at least 10-year duration and without established CVD (CAD, cerebrovascular accident or peripheral artery disease) were included in the study. Subjects with T1DM were consecutively recruited from our outpatient clinic. Exclusion criteria included: i) chronic kidney disease with renal failure (estimated glomerular filtration rate (CKD-EPI) <60ml/min/1.73 m^2^), ii) any other acute/chronic condition associated with an inflammatory response (e.g., acute or chronic inflammatory or infectious diseases), iii) use of anti-inflammatory drugs in the previous 6 months, iv) malignancy disease in the previous 5 years (except basal cell carcinoma), v) hospitalization in the previous 2 months, vi) arrhythmia (except atrial premature complex) and vii) pregnancy. The study protocol was approved by our hospital ethics committee (Parc Taulí Ethics Comitee) and conducted in accordance with the Declaration of Helsinki. All subjects gave their written informed consent before participating in the study.

### Study design

All subjects underwent standardized anamnesis and physical examination. The following information was recorded using a predefined standardized form: age, sex, diabetes duration, family history of premature CVD (defined as CVD occurring before the age of 55 in male and 65 in female first-degree relatives), physical activity (International Physical Activity Questionnaire)[20], active smoking, alcohol intake, insulin dose and the use of any other medication. Body weight, height, and waist and hip circumferences were registered. Systolic and diastolic blood pressure (SBP and DBP, respectively) were measured and mean arterial pressure (MAP) was calculated as 1/3 SBP + 2/3 DBP. After overnight fasting, venous blood samples were taken and complete blood counts, fasting plasma glucose, HbA_1c_, creatinine and lipid profile were determined. Hypertension was defined as having BP>140/90 [21] and/or taking antihypertensive drugs. Dyslipidaemia was defined as having concentrations of total cholesterol >5.2mmol/L, triglycerides >1.7mmol/L, HDL cholesterol <1.03mmol/L, LDL- cholesterol >3.4mmol/L [22] and/or receiving drug treatment for dyslipidaemia.

#### Laboratory analyses

HbA_1c_ was determined by high-performance liquid chromatography (Menarini Diagnostics, Firenze, Italy). Total serum cholesterol, triglycerides and HDL cholesterol were measured using standard enzymatic methods. LDL cholesterol was estimated through the Friedewald formula [23].

#### Metabolic syndrome and insulin-resistance

The metabolic syndrome was assessed according to each of the following three definitions: the National Cholesterol Education Program (NCEP) Adult Treatment Panel III (ATP III), as modified by the AHA/National Heart, Lung, and Blood Institute [24]; the International Diabetes Federation (IDF) [25]; and the World Health Organization (WHO) [26].

To estimate insulin resistance, we used the formula proposed by Williams *et al* for patients with T1DM, subsequently adapted for the use of HbA_1c_ rather than HbA_1_ by Kilpatrick *et al* for its use in the DCCT/EDIC cohort [17,27]. It yields an estimate of the glucose disposal rate (eGDR), taking into account glycaemic control, waist-to-hip ratio (WHR) and blood pressure (eGDR = 24.31–12.22*(WHR)–3.29*(Hypertension 0 = No; 1 = Yes)–0.57*(HbA_1c_))[17]. The formula was validated against euglycemic-hyperinsulinemic clamp in a group of patients with T1DM clinically similar to the subjects evaluated in the current study. Lower eGDR values reflect higher insulin-resistance levels.

#### Assessment of microvascular complications

Peripheral polyneuropathy was assessed through a previously described two-step protocol combining the 15–item MNSI (Michigan Neuropathy Screening Instrument) questionnaire and a physical examination [28]. Retinopathy was always evaluated by the same ophthalmologist. Subjects were classified into three groups according to the degree of retinopathy: no retinopathy, non-proliferative retinopathy or proliferative retinopathy. Nephropathy was assessed by the measurement of urinary albumin/creatinine ratio (ACR). Subjects with an urinary ACR greater than 3.4 mg/mmol [29], or previously treated with converting enzyme inhibitors or angiotensin receptor blockers (for microalbuminuria or macroalbuminuria), were considered as having diabetic nephropathy.

#### Measurement of arterial stiffness

Arterial stiffness (AS) is an early sign of atherosclerosis [30]. In several populations, AS predicts cardiovascular events independently of classical cardiovascular risk factors [31]. Aortic pulse wave velocity (aPWV) is the gold standard for measuring AS [32]. We measured aPWV according to the recommendations of a recent international consensus [32]. The method has been previously described in detail [33]. In brief, aPWV was determined by sequential applanation tonometry using a Millar tonometer (SPC-301, Millar Instruments, Houston, TX, USA) at the carotid and femoral arteries, gated to a three-lead electrocardiography (ECG) using the SphygmoCor^®^ system (AtCor Medical Pty Ltd, West Ryde (Sydney), NSW, Australia). Those aPWV recordings not satisfying the automatic quality controls specified by the SphygmoCor^®^ software were rejected. The mean of two aPWV measurements was taken for each subject for all calculations. Data were available for all the participants included in the study.

#### SMI assessment

All patients were screened for SMI with rest/stress myocardial perfusion imaging (MPI) using 99m-technetium Tetrofosmin single-photon emission computed tomography (SPECT). One-day protocol gated SPECT was used: a first endovenous dose of 370 MBq, administered at rest (30 minutes before image acquisition) and a second dose of 1110 MBq, given at the point of maximum effort on the treadmill exercise.

Imaging acquisition was performed by a Siemens ECAM dual head 90° gamma camera with a low energy high-resolution collimator and a 180° semi-circular orbit, with images every 3 degrees. Acquisition was synchronized with the electrocardiogram R-wave, with an 8-frame/cardiac cycle. Images were reconstructed using filtered black-projection. The calculation of left ventricular ejection fraction and ventricular volumes were automatically assessed with the quantitative software QGS^®^ (Cedars Sinai Medical Centre, Los Angeles, CA). Quantitative and qualitative analyses were performed. To quantify perfusion, the left ventricle was divided into 17 segments, each scored from 0 to 4 (0 = normal perfusion, 1 = mild hypo-perfusion, 2 = moderate hypo-perfusion, 3 = severe hypo-perfusion, and 4 = no perfusion). The summed rest score and the summed stress score were obtained, with the summed score difference as the difference between the two. Myocardial ischemia was defined as showing a summed score difference (SSD) ≥ 2. Three levels of ischemia were considered: mild (SSD = 2–3), moderate (SSD = 4–6), and severe (SSD>7). Asymptomatic patients with abnormal ECG stress test and/or myocardial perfusion defects (SSD> = 2) were diagnosed with SMI.

### Models

In order to assess the suitability of the current recommendations for performing additional testing for CAD (see above), our data were used to calculate the 10-year probability of CAD according to the Framingham Risk Equation [34], the UKPDS Risk Engine [35] and the equation developed in the EDC cohort (specifically designed for patients with T1DM and validated in the cohort from the EURODIAB Prospective Complications Study)[36].

### Statistical analyses

All data were tested for normality using the Shapiro-Wilk test. Data are presented as percentage, mean (SD) for normally distributed quantitative variables, or median (interquartile range) for non-normally distributed quantitative variables. Differences between groups (patients with SMI vs. patients without SMI) were analysed using the χ^2^ test for comparisons of proportions, and the unpaired t-test or the Mann-Whitney U test for comparisons of normally and non-normally distributed quantitative variables, as needed. To identify the factors independently related to SMI, backward stepwise logistic regression analyses were performed. All variables associated in the univariate analyses (0.67<OR>1.67 and p<0.2) and those variables known or likely to be associated with SMI (based on previous literature) were included in those logistic regression models as potential independent variables. Non-normally distributed quantitative variables were used after performing a log_10_-transformation. Receiver-operating characteristic (ROC) curves were developed to represent the prediction of SMI (based on the equations obtained from calculated logistic regression models and on the Framingham, UKPDS and EDC risk scores), in which sensitivity is plotted as a function of 1-specificity. Subsequently, the equality between the different ROC areas obtained was tested. To test the potential relationship between the degree of SMI and the eGDR, an ordered logistic regression model with SMI as the dependent variable and eGDR as the independent one was used. Two-tailed p-values <0.05 were considered statistically significant. The calculations were made using STATA v.13.1 for Mac (StataCorp LP, College Station, TX).

## Results

SMI was diagnosed in 10 out of 84 (11.9%) patients with T1DM (7 with mild, 2 with moderate and 1 with severe ischemia). The main clinical and analytical characteristics of the study population are shown in Table 1. Patients with T1DM and SMI, as compared with those without SMI, were more hypertensive (70.0% vs. 36.5%; p = 0.044), had more insulin resistance (5.5 (4.8–6.7) mg·kg^-1^·min^-1^ vs. 8.1 (5.9–9.5) mg·kg^-1^·min^-1^; p = 0.010) and had a tendency toward a worse glycaemic control (HbA_1c_: 8.3 (7.9–9.4)% vs. 7.7 (7.1–8.6)%; p = 0.053) although it did not reach statistical significance. There were no significant differences between groups regarding other traditional cardiovascular risk factors (such as age, gender, smoking habit, dyslipidaemia or family history of premature CVD) or the prevalence of metabolic syndrome. There were no significant differences for aPWV between groups (p = 0.885). In the univariate analyses, SMI was associated with SBP (OR = 1.062, p = 0.049), HbA_1c_ (OR = 1.936, p = 0.050) and eGDR (OR = 0.671, p = 0.016) (Table 2). In addition, there was an inverse relationship between the degree of SMI and eGDR values (OR = -0.435; p = 0.013).

**Table 1:** Clinical and metabolic characteristics of patients with type 1 diabetes.

	Total (n = 84)	No SMI (n = 74)	SMI (n = 10)	p
***Clinical characteristics***				
Age (yrs.)	50.1 (9.3)	50.0 (9.4)	50.5 (9.0)	0.825
Gender (male/female), n	42/42	35/39	7/3	0.172
Current smokers, n (%)	31.0 (36.9)	25.0 (33.8)	6.0 (60.0)	0.264
Alcohol intake (g/day)	1.4 (0.0–5.0)	1.1 (0–4.3)	2.9 (0–7.1)	0.542
Physical activity (MET-min/s)	1386 (693–2286)	1386 (693–2079)	1386 (924–3093)	0.551
Family history of premature CVD, n (%)	14.0 (16.7)	12.0 (16.2)	2.0 (20.0)	0.768
Family history of T2DM, n (%)	23.0 (27.4)	20.0 (27.0)	3.0 (30.0)	0.844
Hypertension, n (%)	34 (40.5)	27 (36.5)	7 (70.0)	0.044
Dyslipidaemia, n (%)	59 (70.2)	50 (67.6)	9 (90.0)	0.112
***Diabetes***				
Diabetes duration (yrs.)	19.0 (15.0–27.5)	19.0 (15.0–27.0)	18.5 (15.0–33.0)	0.857
Total insulin doses (UI/kg·day)	0.60 (0.53–0.72)	0.60 (0.53–0.73)	0.63 (0.52–0.72)	0.978
Microvascular complications, n (%)	43 (51.2)	36 (48.7)	7 (70.0)	0.199
Retinopathy, n (%)				0.500
- None, n (%)	59 (70.2)	53 (71.6)	6 (60.0)	
- Non-proliferative, n (%)	13 (15.5)	12 (16.2)	1 (10.0)	
- Proliferative, n (%)	12 (14.3)	9 (12.2)	3 (30.0)	
Nephropathy, n (%)	27 (32.1)	23 (31.1)	4 (40.0)	0.577
Peripheral neuropathy, n (%)	5 (6.0)	3 (4.1)	2 (20.0)	0.095
***Anthropometric measurements***				
Weight (kg)	71.8 (13.5)	72.3 (13.4)	68.4 (15.2)	0.400
BMI (kg/m^2^)	26.0 (4.2)	26.2 (4.3)	24.5 (3.3)	0.384
Waist-to-hip ratio	0.91 (0.85–0.96)	0.90 (0.84–0.95)	0.91 (0.90–1.02)	0.186
***Blood pressure***				
SBP (mmHg)	126.4 (12.4)	125.4 (11.8)	133.8 (14.7)	0.045
DBP (mmHg)	71.9 (9.1)	71.7 (8.6)	73.6 (12.8)	0.544
MAP (mmHg)	90.1 (9.3)	89.6 (8.6)	93.7 (13.0)	0.197
***Laboratory parameters***				
White blood cells	6.1 (5.3–7.5)	5.8 (5.2–7.5)	6.5 (6.3–7.3)	0.122
Fasting plasma glucose (mmol/L)	7.4 (5.1–10.6)	7.1 (5.0–10.1)	8.3 (7.7–11.8)	0.266
HbA_1c_ (%)	7.9 (7.1–8.7)	7.7 (7.1–8.6)	8.3 (7.9–9.4)	0.053
HbA_1c_ (mmol/mol)	62 (54–72)	61 (54–71)	67 (63–79)	0.053
Urinary ACR (mg/mmol)	5.1 (3.2–12.5)	4.8 (3.1–12.5)	6.3 (4.3–16.6)	0.407
Total cholesterol (mmol/L)	4.6 (4.2–5.2)	4.6 (4.2–5.2)	4.7 (4.5–5.5)	0.595
HDL-cholesterol (mmol/L)	1.7 (1.4–2.2)	1.8 (1.4–2.2)	1.7 (1.3–1.9)	0.320
LDL-cholesterol (mmol/L)	2.4 (2.1–2.9)	2.4 (2.1–2.8)	3.6 (2.4–3.1)	0.281
Triglycerides (mmol/L)	0.7 (0.6–1.3)	0.7 (0.6–0.8)	0.8 (0.7–1.8)	0.423
***Metabolic syndrome and Insulin resistance***				
Metabolic syndrome				
- NCEP/ATPIII modified	26 (31.0)	23 (31.1)	3 (30.0)	1.000
- IDF definition	30 (35.7)	25 (33.8)	5 (50.0)	0.484
- WHO definition	27 (32.1)	22 (29.7)	5 (50.0)	0.279
eGDR (mg·kg^-1^·min^-1^)	7.8 (5.5–9.4)	8.1 (5.9–9.5)	5.5 (4.8–6.7)	0.010
***Arterial stiffness***				
aPWV (m/s)	7.9 (6.9–9.1)	7.9 (7.0–8.9)	8.1 (6.8–9.4)	0.885

Data are given as percentages, mean (SD) or median (interquartile range). CVD: Cardiovascular disease. T2DM: type 2 diabetes. BMI: body mass index. WHR: waist-to-hip ratio. SBP: systolic blood pressure. DBP: diastolic blood pressure. MAP: mean arterial pressure. ACR: Urinary albumin to creatinine ratio. NCEP/ATPIII: National Cholesterol Education Program/Adult Treatment Panel III. IDF: International Diabetes Federation. WHO: World Health Organization. eGDR: estimation of glucose disposal rate. aPWV: aortic pulse wave velocity.

**Table 2:** Unadjusted odds ratio for the presence of SMI (univariate analysis).

*Variables*	OR (95% CI)	*p*
Age (yrs.)	1.005 (0.936–1.078)	0.890
Gender	0.385 (0.092–1.603)	0.190
Smoking habit	1.678 (0.766–3.676)	0.196
Physical activity	1.000 (0.999–1.000)	0.227
Family history of premature CVD	1.292 (0.244–6.869)	0.764
Family history of T2DM	1.157 (0.272–4.916)	0.843
Hypertension	4.062 (0.969–17.022)	0.055
Dyslipidaemia	4.320 (0.517–36.082)	0.177
Diabetes duration (yrs.)	1.020 (0.949–1.096)	0.589
Total insulin doses (UI/kg·day)	0.358 (0.012–11.160)	0.559
Microvascular complications, n (%)	2.463 (0.591–10.264)	0.216
Retinopathy		
- Non-proliferative	0.736 (0.081–6.695)	0.786
- Proliferative	2.944 (0.621–13.951)	0.174
Nephropathy	1.478 (0.380–5.746)	0.573
Peripheral neuropathy	5.917 (0.856–40.874)	0.071
Weight (kg)	0.977 (0.927–1.031)	0.396
BMI (kg/m^2^)	0.894 (0.745–1.074)	0.231
Waist	0.993 (0.940–1.050)	0.807
SBP (mmHg)	1.062 (1.000–1.128)	0.049
DBP (mmHg)	1.023 (0.952–1.098)	0.539
MAP (mmHg)	1.049 (0.976–1.128)	0.196
Fasting plasma glucose (mmol/L)	1.004 (0.995–1.013)	0.370
HbA_1c_ (%)	1.936 (1.000–3.747)	0.050
Urinary ACR (mg/mmol)	0.997 (0.981–1.013)	0.711
Total cholesterol (mmol/L)	1.007 (0.989–1.026)	0.426
HDL-cholesterol (mmol/L)	0.982 (0.948–1.018)	0.325
LDL-cholesterol (mmol/L)	1.107 (0.996–1.039)	0.112
Triglycerides (mmol/L)	1.003 (0.991–1.016)	0.608
Metabolic syndrome		
- NCEP/ATPIII modified	0.950 (0.225–4.008)	0.945
- IDF definition	1.920 (0.508–7.263)	0.337
- WHO definition	2.364 (0.621–8.991)	0.207
eGDR (mg·kg^-1^·min^-1^)	0.671 (0.485–0.928)	0.016
aPWV (m/s)	1.111 (0.838–1.474)	0.464

OR: Odds ratio. 95% CI: 95% confidence interval. CVD: Cardiovascular disease. T2DM: type 2 diabetes. BMI: body mass index. SBP: systolic blood pressure. DBP: diastolic blood pressure. MAP: mean arterial pressure. ACR: Urinary albumin to creatinine ratio. NCEP/ATPIII: National Cholesterol Education Program/Adult Treatment Panel III. IDF: International Diabetes Federation. WHO: World Health Organization. eGDR: estimation of glucose disposal rate. aPWV: aortic pulse wave velocity.

To evaluate the independent factors associated with SMI, backward stepwise logistic regression models were calculated. The best logistic regression model obtained showed that just two variables, eGDR (OR = -0.593, p = 0.005) and active smoking (OR = 7.964, p = 0.018), were independently associated with SMI, after adjusting for potential confounders (Table 3). The ROC area under the curve (AUC) for the model including eGDR and active smoking was 0.833 (95% confidence interval (CI): 0.692–0.974). The best cut-off point derived from the ROC curve showed a sensitivity of 70% and specificity of 86.3% and correctly classified 84.3% of patients (corresponding to an eGDR = 3.836 for non-smokers and eGDR = 7.338 for active-smokers).

**Table 3:** Best multiple logistic regression model for predicting silent myocardial ischemia.

LR χ^2^ 13.19, *p* = 0.001	Coefficient	SE	(95% CI)	OR	*p*
eGDR	-0.593	0.211	-1.006 - -0.179	0.553	0.005
Current smokers	2.074	0.880	0.350–3.799	7.964	0.018
Constant	0.909	1.147	-1.338–3.157	2.483	0.428

Dependent variable: silent myocardial ischemia. Independent variables initially included in the model: estimation of glucose disposal rate (eGDR), age, sex (male/female), BMI, smoking habit, dyslipidaemia (No/Yes), diabetic retinopathy and peripheral neuropathy (No/Yes).

**Fig 1:**
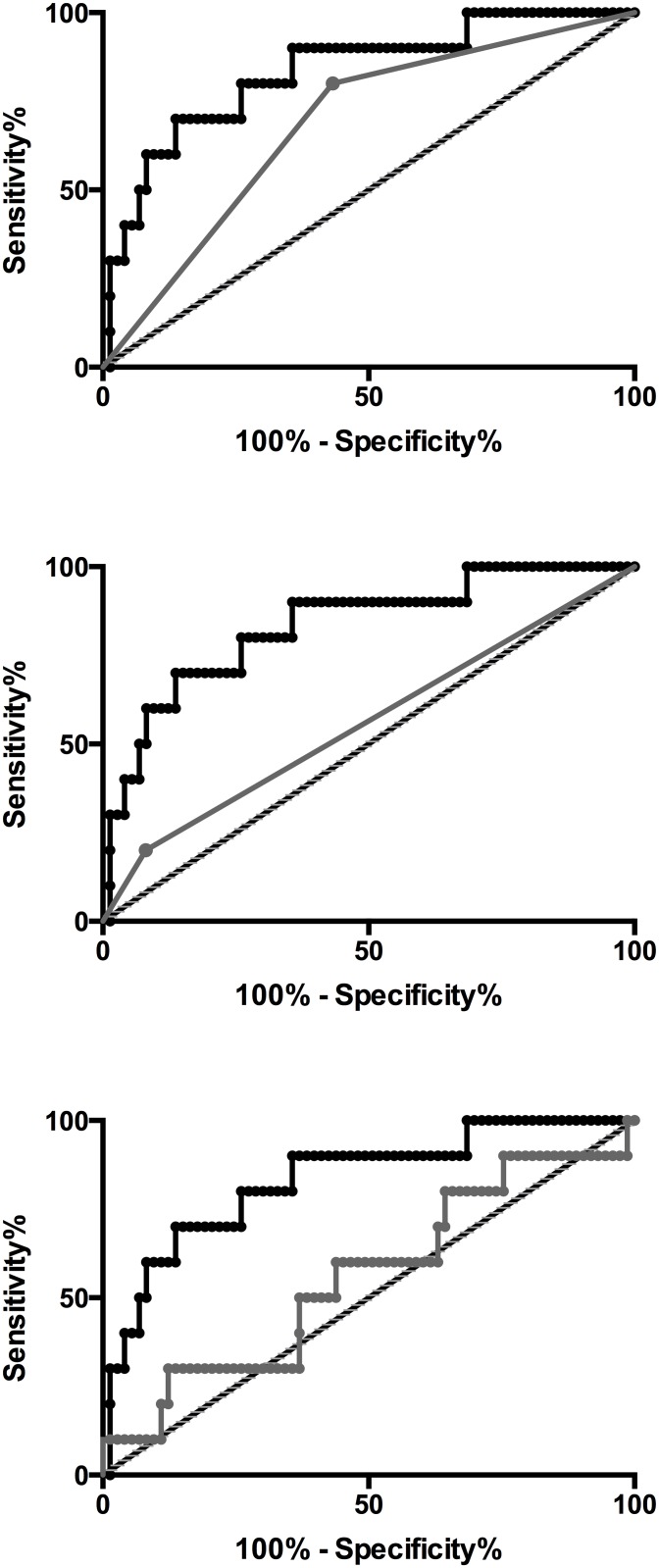
ROC curves to detect Silent Myocardial Ischemia (SMI). The area under the curve (AUC) of the three risk scores tended to underestimate SMI risk when compared with our proposed model. **1A)** Comparison with the Framingham Risk Score [0.833 (95% CI: 0.692–0.974) vs. 0.688 (95% CI: 0.545–0.830, p = 0.122)]. **1B)** Comparison with the UKPDS Risk Score [0.833 (95% CI: 0.692–0.974) vs. 0.559 (95% CI: 0.424–0.693), p = 0.001]. **1C)** Comparison with the EDC Risk Score [0.833 (95% CI: 0.692–0.974) vs. 0.558 (95% CI: 0.352–0.763), p = 0.027].

To assess the accuracy of the previous existing risk scores for predicting clinical CAD events in the setting of the SMI prediction, Framingham, UKPDS and EDC risk scores were used and their ROC curves were developed. The AUCs for the prediction of SMI for each of these ROC curves were 0.688 (95% CI: 0.545–0.830) for the Framingham risk score, 0.559 (95% CI: 0.424–0.693) for the UKPDS and 0.558 (95% CI: 0.352–0.763) for the EDC. All three equations, when compared with our model, underestimate the SMI risk, non-significantly in the case of the Framingham risk score (0.833 vs. 0.688, p = 0.122) but significantly in the case of the UKPDS (0.833 vs. 0.559, p = 0.001) and EDC risk scores (0.833 vs. 0.558, p = 0.027)(Fig 1).

## Discussion

The present study provides, for the first time, a good, sensitive risk-estimation model for predicting SMI in T1DM. Furthermore, it also shows that SMI (detected by stress MPI-SPECT tests) is relatively common in patients with T1DM of at least ten-year duration and no previous clinical cardiovascular disease and that it is associated with active smoking and insulin-resistance in this population. These results have the potential to lead to improvements in CAD care in T1DM through a strategy focused on accurate, cost-effective detection of SMI.

Studies evaluating the prevalence of SMI in subjects with T1DM are scarce and differ in both the clinical characteristics of patients included and the techniques used to evaluate SMI. Larsen *et al* found a prevalence of SMI of 15% using exercise ECG in 45 asymptomatic patients with T1DM and without macro-vascular complications [9]. Sultan *et al*. described a prevalence of 22% using stress MPI tests in 135 asymptomatic patients with T1DM (but including patients with peripheral artery disease)[11], while in the study performed by Senior *et al*. SMI (assessed by coronary angiographies) was diagnosed in 30 out of 53 T1DM candidates for islet transplantation (56.6%)[10]. In our study we found a prevalence of 11.9% in 84 patients with T1DM and without clinical CVD. All these results confirm a higher prevalence of SMI in patients with T1DM compared with the general population, where it is considered to affect 1–4% of subjects [12]. However, at the moment there are no specific recommendations for SMI screening in T1DM. Consequently, the ADA/AHA guidelines recommend performing additional CAD testing on any patient (including those with T1DM) based on global risk scores derived from general or T2DM population cohorts [34]. This strategy is based on the fact that no previous study has demonstrated that the use of any of the available tests for detecting SMI was cost-effective in T2DM [37]. Nevertheless, this global strategy has several flaws, which could hamper any significant advance in CAD care in diabetes, especially in T1DM. Firstly, these models do not identify a substantial proportion of T1DM patients at highest risk of CAD events. In the Pittsburgh EDC cohort, the use of Framingham Heart Study and UKPDS algorithms did not demonstrate good predictive results, underestimating the probability of CAD events in the highest risk deciles [38]. Secondly, and probably more importantly, these models were designed for predicting clinical CAD events, but not the silent ones. However, in the DCCT/EDIC study, half of non-fatal MIs were silent [8]. Thus, any novel risk estimation model aimed at really improving CAD care in T1DM should not overlook this important clinical fact.

The current study provides the first risk estimation model ever developed for predicting SMI in T1DM subjects. This model significantly enhanced our capacity to detect T1DM patients with SMI compared with current risk estimation models for predicting clinical CAD events in people with diabetes. Our model, which combines only two clinical variables (active smoking and insulin-resistance), correctly classified more than eight out of ten of these subjects and significantly improved the results obtained from the use of the current available risk estimation models designed for predicting clinical CAD events in diabetes, such as the UKPDS Risk Engine (T2DM) or the equation derived from the Pittsburgh EDC Study (T1DM).

Interestingly, the main factors associated with SMI in our cohort were active smoking and insulin resistance (measured as eGDR). It is well known that smoking is a major risk factor for CVD. In T1DM, smoking has been associated with several cardiovascular risk factors (such as physical inactivity, worse glycaemic control or more atherogenic lipid profile and endothelial dysfunction)[39,40]. The role of smoking as cardiovascular risk factor in T1DM is strongly supported by a recent risk estimation model for predicting clinical cardiovascular events (a composite of fatal and non-fatal events of CAD, ischemic stroke, heart failure and peripheral artery disease) developed in a large cohort of T1DM subjects, in which smoking was identified as one of its main predictor factors [41]. However, there are no previous studies evaluating the effects of smoking on SMI in T1DM.

Although T1DM is characterized by insulin deficiency, insulin resistance is also a common finding in patients with T1DM, a condition known as double diabetes [42,43]. In our study, the prevalence of metabolic syndrome ranged from 31% (NCEP-ATPIII) to 36% (IDF), similar to previous results [44]. However, we do not find any association between SMI and the three metabolic syndrome definitions assessed. Nonetheless, the current study shows an association between insulin resistance (measured as eGDR) and SMI for the first time in the literature. In accordance with these results, eGDR has been previously associated with the prediction of clinical CAD events in subjects with T1DM from either the Pittsburgh EDC cohort or the DCCT/EDIC cohort [16–18]. These data are in line with our initial hypothesis. In addition, the finding that an inverse association exists between the degree of SMI and eGDR, reinforces the potential role of eGDR for predicting SMI. Consequently, insulin-resistance, assessed as eGDR, would be an important factor in identifying those patients with T1DM at highest risk of SMI.

Microangiopathy has been traditionally linked to CVD and it has been suggested that both micro and macrovascular complications share common pathogenic mechanisms [45]. However, we did not find any significant association between SMI and microvascular complications. Studies evaluating the likely relationship between SMI in T1DM and microvascular complications are rare. We only have found the study from Sultan *et al* in which an association between SMI and microangiopathy (defined as the presence of albuminuria or diabetic retinopathy) was described, although they did not find any association when each component of the microangiopathy was analysed separately [11]. In contrast with the study of Sultan *et al*, we did not include patients with clinical CVD and our patients had a better glycaemic control, an important factor contributing to lower the prevalence of microvascular complications. In fact, the number of microvascular complications (nephopathy, retinopathy and peripheral neuropathy) was so small that the study was underpowered to evaluate such an association.

In the current study, the lack of association between SMI and T1DM duration deserves further comment. To our knowledge, no previous study has evaluated this likely association. Our study just included those persons with T1DM duration of more than 10 years. This fact lowers the range of T1DM duration and it may justify the described lack of association between SMI and T1DM duration. Thus, the design of the current study was not the most appropriate to evaluate this issue.

The major limitation of the current study is its cross-sectional design, which makes it impossible to determine the temporal ordering of the association between SMI and active smoking or insulin resistance. Nonetheless, it seems reasonable to think that both factors might be involved in the pathogenesis of SMI. In addition, the study was observational in design and consequently complete control of all potential (unknown) confounding factors could not be ensured.

In summary, the current study indicates a relatively high prevalence of SMI in subjects aged 35–65 years with T1DM of at least 10-year duration and no previous clinical cardiovascular disease. Additionally, in these subjects, both active smoking and insulin resistance were independently associated with SMI. Finally, the study reports the first SMI-risk estimation model in T1DM, which could be of great utility in better identification of those individuals at higher risk of SMI. Nevertheless, further studies are needed to validate these results in larger cohorts, to improve the model’s accuracy with the addition of novel cardiovascular biomarkers and to test its cost-effectiveness in routine clinical practice.

## Supporting information

S1 Data Dataset file. (DTA)

## References

[1] Libby P, Nathan DM, Abraham K, Brunzell JD, Fradkin JE, Haffner SM, et al (2005) Report of the National Heart, Lung, and Blood Institute-National Institute of Diabetes and Digestive and Kidney Diseases Working Group on Cardiovascular Complications of Type 1 Diabetes Mellitus. Circulation 111: 3489–3493. 10.1161/CIRCULATIONAHA.104.529651 15983263

[2] Laing SP, Swerdlow AJ, Slater SD, Burden AC, Morris A, Waugh NR, et al (2003) Mortality from heart disease in a cohort of 23,000 patients with insulin-treated diabetes. Diabetologia 46: 760–765. 10.1007/s00125-003-1116-6 12774166

[3] Secrest AM, Becker DJ, Kelsey SF, Laporte RE, Orchard TJ (2010) Cause-specific mortality trends in a large population-based cohort with long-standing childhood-onset type 1 diabetes. Diabetes 59: 3216–3222. 10.2337/db10-0862 20739685 PMC2992785

[4] de Ferranti SD, de Boer IH, Fonseca V, Fox CS, Golden SH, Lavie CJ, et al (2014) Type 1 diabetes mellitus and cardiovascular disease: a scientific statement from the American Heart Association and American Diabetes Association. Diabetes Care 37: 2843–2863. 10.2337/dc14-1720 25114297 PMC4170130

[5] Orchard TJ, Costacou T, Kretowski A, Nesto RW (2006) Type 1 diabetes and coronary artery disease. Diabetes Care 29: 2528–2538. 10.2337/dc06-1161 17065698

[6] Livingstone SJ, Levin D, Looker HC, Lindsay RS, Wild SH, Joss N, et al (2015) Estimated life expectancy in a Scottish cohort with type 1 diabetes, 2008–2010. JAMA 313: 37–44. 10.1001/jama.2014.16425 25562264 PMC4426486

[7] Fava S, Azzopardi J, Muscat HA, Fenech FF (1993) Factors that influence outcome in diabetic subjects with myocardial infarction. Diabetes Care 16: 1615–1618. 8299458 10.2337/diacare.16.12.1615

[8] Diabetes C, Complications Trial /Epidemiology of Diabetes I, Complications Study Research G (2016) Intensive Diabetes Treatment and Cardiovascular Outcomes in Type 1 Diabetes: The DCCT/EDIC Study 30-Year Follow-up. Diabetes Care 39: 686–693. 10.2337/dc15-1990 26861924 PMC4839174

[9] Larsen J, Brekke M, Sandvik L, Arnesen H, Hanssen KF, Dahl-Jorgensen K (2002) Silent coronary atheromatosis in type 1 diabetic patients and its relation to long-term glycemic control. Diabetes 51: 2637–2641. 12145181 10.2337/diabetes.51.8.2637

[10] Senior PA, Welsh RC, McDonald CG, Paty BW, Shapiro AM, Ryan EA (2005) Coronary artery disease is common in nonuremic, asymptomatic type 1 diabetic islet transplant candidates. Diabetes Care 28: 866–872. 15793187 10.2337/diacare.28.4.866

[11] Sultan A, Piot C, Mariano-Goulart D, Rasamisoa M, Renard E, Avignon A (2004) Risk factors for silent myocardial ischemia in high-risk type 1 diabetic patients. Diabetes Care 27: 1745–1747. 15220258 10.2337/diacare.27.7.1745

[12] Fazzini PF, Prati PL, Rovelli F, Antoniucci D, Menghini F, Seccareccia F, et al (1993) Epidemiology of silent myocardial ischemia in asymptomatic middle-aged men (the ECCIS Project). Am J Cardiol 72: 1383–1388. 8256731 10.1016/0002-9149(93)90184-e

[13] Cosson E, Guimfack M, Paries J, Paycha F, Attali JR, Valensi P (2003) Prognosis for coronary stenoses in patients with diabetes and silent myocardial ischemia. Diabetes Care 26: 1313–1314. 12663617 10.2337/diacare.26.4.1313

[14] Cosson E, Guimfack M, Paries J, Paycha F, Attali JR, Valensi P (2003) Are silent coronary stenoses predictable in diabetic patients and predictive of cardiovascular events? Diabetes Metab 29: 470–476. 14631323 10.1016/s1262-3636(07)70060-5

[15] Valensi P, Paries J, Brulport-Cerisier V, Torremocha F, Sachs RN, Vanzetto G, et al (2005) Predictive value of silent myocardial ischemia for cardiac events in diabetic patients: influence of age in a French multicenter study. Diabetes Care 28: 2722–2727. 16249546 10.2337/diacare.28.11.2722

[16] Orchard TJ, Olson JC, Erbey JR, Williams K, Forrest KY, Smithline Kinder L, et al (2003) Insulin resistance-related factors, but not glycemia, predict coronary artery disease in type 1 diabetes: 10-year follow-up data from the Pittsburgh Epidemiology of Diabetes Complications Study. Diabetes Care 26: 1374–1379. 12716791 10.2337/diacare.26.5.1374

[17] Kilpatrick ES, Rigby AS, Atkin SL (2007) Insulin resistance, the metabolic syndrome, and complication risk in type 1 diabetes: "double diabetes" in the Diabetes Control and Complications Trial. Diabetes Care 30: 707–712. 10.2337/dc06-1982 17327345

[18] Pambianco G, Costacou T, Orchard TJ (2007) The prediction of major outcomes of type 1 diabetes: a 12-year prospective evaluation of three separate definitions of the metabolic syndrome and their components and estimated glucose disposal rate: the Pittsburgh Epidemiology of Diabetes Complications Study experience. Diabetes Care 30: 1248–1254. 10.2337/dc06-2053 17303788

[19] Diabetes C, Complications Trial/Epidemiology of Diabetes I, Complications Research G (2016) Risk Factors for Cardiovascular Disease in Type 1 Diabetes. Diabetes 65: 1370–1379. 10.2337/db15-1517 26895792 PMC4839209

[20] Hallal PC, Victora CG (2004) Reliability and validity of the International Physical Activity Questionnaire (IPAQ). Med Sci Sports Exerc 36: 556 15076800 10.1249/01.mss.0000117161.66394.07

[21] Mancia G, De Backer G, Dominiczak A, Cifkova R, Fagard R, Germano G, et al (2007) 2007 Guidelines for the management of arterial hypertension: The Task Force for the Management of Arterial Hypertension of the European Society of Hypertension (ESH) and of the European Society of Cardiology (ESC). Eur Heart J 28: 1462–1536. 10.1093/eurheartj/ehm236 17562668

[22] National Cholesterol Education Program Expert Panel on Detection E, Treatment of High Blood Cholesterol in A (2002) Third Report of the National Cholesterol Education Program (NCEP) Expert Panel on Detection, Evaluation, and Treatment of High Blood Cholesterol in Adults (Adult Treatment Panel III) final report. Circulation 106: 3143–3421. 12485966

[23] Friedewald WT, Levy RI, Fredrickson DS (1972) Estimation of the concentration of low-density lipoprotein cholesterol in plasma, without use of the preparative ultracentrifuge. Clin Chem 18: 499–502. 4337382

[24] Grundy SM, Cleeman JI, Daniels SR, Donato KA, Eckel RH, Franklin BA, et al (2005) Diagnosis and management of the metabolic syndrome: an American Heart Association/National Heart, Lung, and Blood Institute Scientific Statement. Circulation 112: 2735–2752. 10.1161/CIRCULATIONAHA.105.169404 16157765

[25] International Diabetes Federation: The IDF consensus worldwide definition of the metabolic syndrome [article online], 2003. http://www.idf.org/webdata/docs/IDF_metasyndrome_definition.pdf. Accessed 15 May 2006.

[26] World Health Organization: Definition, Diagnosis and Classification of Diabetes Mellitus and Its Complications: Report of aWHO Consultation. Geneva, World Health Org., Department of Noncommunicable Disease Surveillance, 1999, p. 31–33.

[27] Williams KV, Erbey JR, Becker D, Arslanian S, Orchard TJ (2000) Can clinical factors estimate insulin resistance in type 1 diabetes? Diabetes 49: 626–632. 10871201 10.2337/diabetes.49.4.626

[28] Gonzalez-Clemente JM, Gimenez-Perez G, Richart C, Broch M, Caixas A, Megia A, et al (2005) The tumour necrosis factor (TNF)-alpha system is activated in accordance with pulse pressure in normotensive subjects with type 1 diabetes mellitus. Eur J Endocrinol 153: 687–691. 10.1530/eje.1.02016 16260427

[29] American Diabetes A (2011) Standards of medical care in diabetes—2011. Diabetes Care 34 Suppl 1: S11–61. 21193625 10.2337/dc11-S011 PMC3006050

[30] Cavalcante JL, Lima JA, Redheuil A, Al-Mallah MH (2011) Aortic stiffness: current understanding and future directions. J Am Coll Cardiol 57: 1511–1522. 10.1016/j.jacc.2010.12.017 21453829

[31] Vlachopoulos C, Aznaouridis K, Stefanadis C (2010) Prediction of cardiovascular events and all-cause mortality with arterial stiffness: a systematic review and meta-analysis. J Am Coll Cardiol 55: 1318–1327. 10.1016/j.jacc.2009.10.061 20338492

[32] Laurent S, Cockcroft J, Van Bortel L, Boutouyrie P, Giannattasio C, Hayoz D, et al (2006) Expert consensus document on arterial stiffness: methodological issues and clinical applications. Eur Heart J 27: 2588–2605. 10.1093/eurheartj/ehl254 17000623

[33] Llaurado G, Ceperuelo-Mallafre V, Vilardell C, Simo R, Freixenet N, Vendrell J, et al (2012) Arterial stiffness is increased in patients with type 1 diabetes without cardiovascular disease: a potential role of low-grade inflammation. Diabetes Care 35: 1083–1089. 10.2337/dc11-1475 22357186 PMC3329819

[34] Greenland P, Alpert JS, Beller GA, Benjamin EJ, Budoff MJ, Fayad ZA, et al (2010) 2010 ACCF/AHA guideline for assessment of cardiovascular risk in asymptomatic adults: executive summary: a report of the American College of Cardiology Foundation/American Heart Association Task Force on Practice Guidelines. Circulation 122: 2748–2764. 10.1161/CIR.0b013e3182051bab 21098427

[35] Stevens RJ, Kothari V, Adler AI, Stratton IM, United Kingdom Prospective Diabetes Study G (2001) The UKPDS risk engine: a model for the risk of coronary heart disease in Type II diabetes (UKPDS 56). Clin Sci (Lond) 101: 671–679. 11724655

[36] Zgibor JC, Ruppert K, Orchard TJ, Soedamah-Muthu SS, Fuller J, Chaturvedi N, et al (2010) Development of a coronary heart disease risk prediction model for type 1 diabetes: the Pittsburgh CHD in Type 1 Diabetes Risk Model. Diabetes Res Clin Pract 88: 314–321. 10.1016/j.diabres.2010.02.009 20236721 PMC2891292

[37] Young LH, Wackers FJ, Chyun DA, Davey JA, Barrett EJ, Taillefer R, et al (2009) Cardiac outcomes after screening for asymptomatic coronary artery disease in patients with type 2 diabetes: the DIAD study: a randomized controlled trial. JAMA 301: 1547–1555. 10.1001/jama.2009.476 19366774 PMC2895332

[38] Zgibor JC, Piatt GA, Ruppert K, Orchard TJ, Roberts MS (2006) Deficiencies of cardiovascular risk prediction models for type 1 diabetes. Diabetes Care 29: 1860–1865. 10.2337/dc06-0290 16873793

[39] Schwab KO, Doerfer J, Hallermann K, Krebs A, Schorb E, Krebs K, et al (2008) Marked smoking-associated increase of cardiovascular risk in childhood type 1 diabetes. Int J Adolesc Med Health 20: 285–292. 19097567 10.1515/ijamh.2008.20.3.285

[40] Reynolds K, Liese AD, Anderson AM, Dabelea D, Standiford D, Daniels SR, et al (2011) Prevalence of tobacco use and association between cardiometabolic risk factors and cigarette smoking in youth with type 1 or type 2 diabetes mellitus. J Pediatr 158: 594–601 e591 10.1016/j.jpeds.2010.10.011 21129757 PMC3908882

[41] Vistisen D, Andersen GS, Hansen CS, Hulman A, Henriksen JE, Bech-Nielsen H, et al (2016) Prediction of First Cardiovascular Disease Event in Type 1 Diabetes Mellitus: The Steno Type 1 Risk Engine. Circulation 133: 1058–1066. 10.1161/CIRCULATIONAHA.115.018844 26888765

[42] Yki-Jarvinen H, Koivisto VA (1986) Natural course of insulin resistance in type I diabetes. N Engl J Med 315: 224–230. 10.1056/NEJM198607243150404 3523247

[43] Cleland SJ (2012) Cardiovascular risk in double diabetes mellitus—when two worlds collide. Nat Rev Endocrinol 8: 476–485. 10.1038/nrendo.2012.47 22488644

[44] Thorn LM, Forsblom C, Fagerudd J, Thomas MC, Pettersson-Fernholm K, Saraheimo M, et al (2005) Metabolic syndrome in type 1 diabetes: association with diabetic nephropathy and glycemic control (the FinnDiane study). Diabetes Care 28: 2019–2024. 16043748 10.2337/diacare.28.8.2019

[45] Orasanu G, Plutzky J (2009) The pathologic continuum of diabetic vascular disease. J Am Coll Cardiol 53: S35–42. 10.1016/j.jacc.2008.09.055 19179216 PMC2663393

